# A Southern Indian Ocean database of hydrographic profiles obtained with instrumented elephant seals

**DOI:** 10.1038/sdata.2014.28

**Published:** 2014-09-02

**Authors:** Fabien Roquet, Guy Williams, Mark A. Hindell, Rob Harcourt, Clive McMahon, Christophe Guinet, Jean-Benoit Charrassin, Gilles Reverdin, Lars Boehme, Phil Lovell, Mike Fedak

**Affiliations:** 1 Department of Meteorology, Stockholm University, S-106 91 Stockholm, Sweden; 2 Antarctic Climate and Ecosystem Cooperative Research Centre, University of Tasmania, Hobart 7001, Australia; 3 Institute for Marine and Antarctic Studies, University of Tasmania, Hobart 7001, Australia; 4 Department of Biological Sciences, Macquarie University, Sydney, NSW 2109, Australia; 5 Sydney Institute for Marine Science, Mosman, NSW 2088, Australia; 6 Centre d'Etudes Biologiques de Chizé, CNRS UMR 7372, 79360 Villiers-en-Bois, France; 7 LOCEAN Sorbonne Universités (UPMC, Univ Paris 6)/CNRS/IRD/MNHN, 75252 Paris, France; 8 Sea Mammal Research Unit, University of St Andrews, St Andrews KY16 8LB, UK

## Abstract

The instrumentation of southern elephant seals with satellite-linked CTD tags has offered unique temporal and spatial coverage of the Southern Indian Ocean since 2004. This includes extensive data from the Antarctic continental slope and shelf regions during the winter months, which is outside the conventional areas of Argo autonomous floats and ship-based studies. This landmark dataset of around 75,000 temperature and salinity profiles from 20–140 °E, concentrated on the sector between the Kerguelen Islands and Prydz Bay, continues to grow through the coordinated efforts of French and Australian marine research teams. The seal data are quality controlled and calibrated using delayed-mode techniques involving comparisons with other existing profiles as well as cross-comparisons similar to established protocols within the Argo community, with a resulting accuracy of ±0.03 °C in temperature and ±0.05 in salinity or better. The data offer invaluable new insights into the water masses, oceanographic processes and provides a vital tool for oceanographers seeking to advance our understanding of this key component of the global ocean climate.

## Background & Summary

The Southern Ocean plays a fundamental role in the global climate system. It is home to the Antarctic Circumpolar Current (ACC), the largest current system in the world, which connects water masses from the global ocean basins^1^. It is also where Antarctic Bottom Water (AABW), the dense water mass found in the abyss of the ocean, is formed^2^. Considerable efforts have been directed towards an improved understanding of the Southern Ocean circulation and its response to global climate change during the last decades. However, these efforts remain greatly limited by the lack of *in situ* measurements.

**Figure 1:**
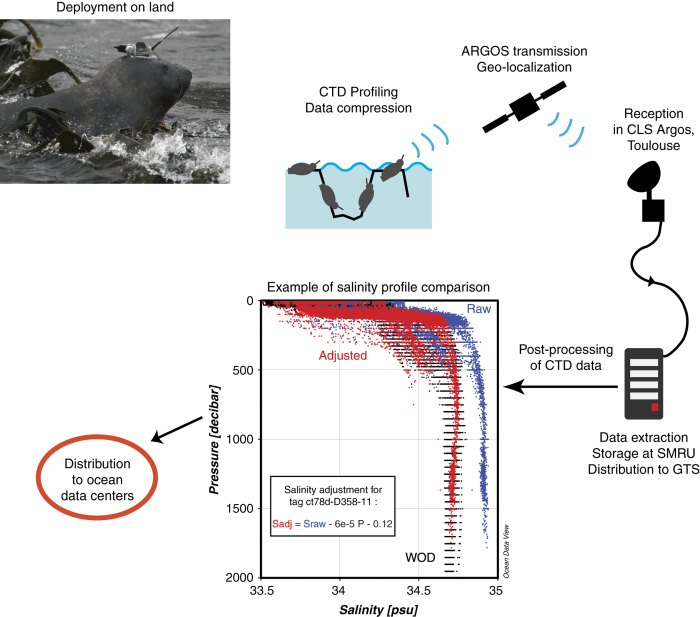
Schematic overview of the procedure used to collect oceanographic data using instrumented elephants seals. The CTD-SRDL is attached to the seal on land, then it records hydrographic profiles during its foraging trips, sending the data by satellite ARGOS whenever the seal goes back to the surface. Position of seals is obtained by triangulation from ARGOS satellites. Data are then extracted, formatted and stored at the Sea Mammal Research Unit (SMRU), and transmitted in real-time to the Global Telecommunication System (GTS). A post-processing procedure is applied on the CTD data, including the editing, adjustment and validation of hydrographic profiles. Finally, data are made available publicly through the British Oceanographic Data Centre (BODC) portal (Data Citation 1).

Over the last decade, southern elephant seals (*Mirounga leonina*) have been instrumented with CTD-SRDL tags (CTD stands for Conductivity-Temperature-Depth, and SRDL for Satellite-Relayed Data Loggers), measuring vertical profiles of temperature and salinity during their foraging trips^2–4,2–4,2–4^ (Fig. 1). While the primary motivation for this work has been to further the understanding of the influence of the oceanographic environment to the foraging success, reproductive performance and population trajectories of the seals^5–10,5–10,5–10,5–10,5–10,5–10^, the unique ability of elephant seals to continuously dive to great depths (mean 590±200 m, with maxima over 2,000 m) for long durations (average length of a dive 25±15 min, maximum 120 min) has generated a large database of hydrographic profiles for the Southern Ocean^11,12^.

Seal-derived data greatly complements other *in situ* data sources, such as ship-based measurements and Argo profilers. The seal contribution is particularly important in the regions south of the ACC, which are the most difficult to observe because of the seasonal presence of sea ice. Using the ECCO (Estimating the Climate and Circulation of the Oceans) framework^13^, it has been demonstrated that seal-derived data are able to significantly improve state estimates of the Southern Ocean circulation, improving in particular the agreement of the estimated sea ice distribution with independent satellite observations^12^. The latter study highlighted the importance of expanding data collection to the under-represented zones under sea ice and on the Antarctic continental shelf.

An especially large amount of seal-derived data has been gathered in the Indian sector of the Southern Ocean, with regular deployments being carried out on the French owned Kerguelen Islands every year since 2004, and intensive deployments on the Antarctic continent from the two Australian stations Davis and Casey in 2011 and 2012 (Table 1). Part of these seal-derived data have been successfully used to improve our knowledge of the ACC structure across the shallow Kerguelen Plateau, providing the first detailed observations of the swift ACC branch flowing over the Kerguelen Plateau across the so-called Fawn Trough^14,15^, as well as complementary information on the water mass distribution over the biologically productive northern Kerguelen Plateau^16^.

The greatest ‘gap-filling’ by the elephant seal data, relative to Argo and ship-based data coverage, is in and around the continental slope and shelf region of Antarctica^17–22,17–22,17–22,17–22,17–22,17–22^ (more than 45% of seal profiles were obtained south of 60 °S). Seals return data in regions and seasons that have never been directly surveyed, advancing new oceanographic knowledge that would otherwise remain undiscovered. Recently, the seal data revealed the existence of very saline shelf waters in the Cape Darnley polynya (68 °E), in a region and season that is inaccessible to traditional ship-based surveys^23^. Seal-based observation of this dense shelf water, together with overflows of cold, dense modified shelf water on the continental slope, was combined with moored observations of newly formed AABW downstream to provide the first evidence for a new, fourth region of AABW production. Another somewhat more modest source of AABW has also been described offshore of Casey station in the Vincennes Bay polynya^24^.

This data descriptor presents the database of hydrographic (i.e., temperature and salinity) profiles obtained by instrumented seals in the Southern Indian Ocean. This database is a subset of the MEOP-CTD database (MEOP: Marine Mammals Exploring the Oceans Pole to Pole)^12^, complemented with the most recent Southern Indian Ocean deployment data. The MEOP-CTD database currently includes more than 200,000 seal-derived hydrographic profiles with near circumpolar distribution, and will continue to grow as more data become available. The Southern Indian Ocean database represents about one third of the entire MEOP-CTD database, with about 75,000 profiles obtained from 207 CTD-SRDL tag deployments (Table 1).

## Methods

The CTD–Satellite Relay Data Loggers (CTD-SRDLs) are built by the Sea Mammal Research Unit (SMRU, University of St Andrews, UK), incorporating CTD sensors developed by Valeport Ltd (Devon, UK). The sensor head consists of a pressure transducer, a platinum resistance thermometer, and an inductive cell for measuring conductivity. The temperature and conductivity sensors have a precision (repeatability) of 0.005 °C and 0.005 mS/cm, respectively. Before being taken into the field, devices are calibrated in the laboratory by Valeport. Some of the CTD-SRDLs (about half) were also tested at sea against a ship-based CTD before the deployment.

**Figure 2:**
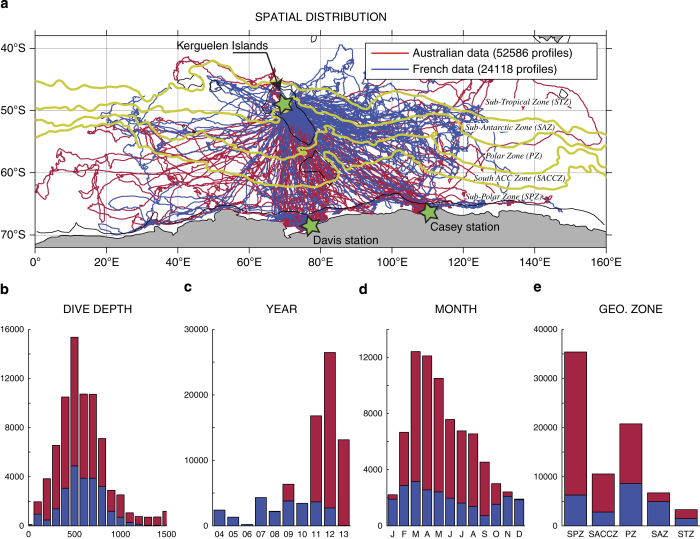
The Southern Indian Ocean database. (**a**) Spatial distribution of seal profiles available in the Southern Indian Ocean and their distribution by (**b**) dive depth, (**c**) year, (**d**) month and (**e**) geographical zone. The position of deployment sites (green stars) and the definition of geographical zones (selected contours of mean dynamic topography^46^ in yellow) are indicated on the spatial distribution map. Australian data (red) were collected through the Integrated Marine Observing System (IMOS), while French data (blue) were obtained in the framework of the observatory MEMO (Mammifères Echantillonneurs du Milieu Marin).

Southern elephant seals (*n*=207) were captured at three locations in the Southern Indian Ocean (Fig. 2 and Supplementary Figure 1), Kerguelen Islands (49.35°S 70.219°E), Davis Station (68°35′ S 77°58′ E) and Casey Station (66°17′ S 110°31′ E). The seals were captured at the end of their annual breeding season (prior to the summer migration) or at the end of their annual moult season (prior to the winter migration). The seals were chemically sedated, weighed and measured, and a CTD-SRDL was attached to the seal's head with two-part epoxy^25^.

The combined weight of each tag and glue is approximately 0.5 kg, i.e., 0.15% and 0.10% of the mean departure weight of adult female southern elephant seals (338±65 kg) and sub-adult males (469±202 kg), respectively. We are confident that the instruments did not affect at-sea behaviour given that the smallest instrumented seal weighed 169 kg (instruments are <0.3% of the seals' weight). Previous studies have demonstrated that seals carrying twice this load (instruments of up to 0.6% of their mass) were unaffected in either the short-term (growth rates) or the long-term (survival) by carrying these instruments^26^. Animal handlings were performed in accordance with relevant guidelines and regulations, after approval by the University of Tasmania and Macquarie University's Animal Ethics Committees for Australian deployments and by the Institut Paul-Emile Victor (IPEV) Ethics Committee for French deployments. The tags are either recovered from the animals when they haul out or they fall off in the subsequent moult, so that a tag can never stay attached on a seal’s head more than 12 months in a row.

CTD-SRDLs record hydrographic profiles during the ascent of seals^27,28^, retaining only the deepest dive in each six-hour time interval, and transmitting profiles in a compressed form (between 10 and 25 data points per profile, depending on the tag program) through the Advanced Research and Global Observation Satellite (ARGOS) system. First guess locations are determined based on the Doppler shifts observed from uplinks. End-of-dive locations are then estimated using a straightforward least-squares method, or a more elaborated Kalman filtering method. The latter method has been developed recently, so it is used for the most recent CTD-SRDLs only (67 tags out of 207). A simple speed filter is then applied to exclude locations that would require an unfeasibly high speed to reach its four neighbouring points (two before and two after), and a linear interpolation is finally applied between the locations that passed the filter^29^. The accuracy of ARGOS geo-positioning is typically about ±5 km^30–33,30–33,30–33,30–33^. The real-time temperature and salinity profile data are made freely available daily via the Global Telecommunication System (GTS) of the World Meteorological Organization (WMO, see www.wmo.int), for immediate use by weather forecasters and ship operators.

Hydrographic profiles are then post-processed using a unified procedure of editing, adjustment, and validation^28^. Each individual CTD-SRDL dataset is post-processed separately, as each tag has different technical specifications and a different life history. A standard set of tests, adapted from Argo standard quality-control procedures^34^, is first run to remove bad profiles, spikes, and outliers. Temperature and salinity adjustments are then determined, which vary from tag to tag, and they are applied identically to all profiles from a given tag.

A salinity adjustment is first estimated for each tag in the form of a pressure dependent linear correction. This salinity bias is known to be primarily induced by an external field effect on the conductivity sensor, which cannot be corrected *a priori* because it depends on how the tag has been attached on the seal’s head^27,28^, and must therefore be corrected using delayed-mode techniques. The error model was suggested by numerous comparisons of CTD-SRDL profiles with ship-based CTD carried out priori to the deployment^28^. For CTD-SRDLs with profiles sampled in frozen areas, a temperature offset is also estimated using the local freezing temperature as a reference. Owing to the relatively short life duration of CTD-SRDLs (typically 4 to 8 months), sensor drifts are assumed to be negligible, which is why the same adjustments are applied to all profiles from a given CTD-SRDL. There are several cases of seals staying in the same region for several months, or returning to a previously visited region, which indicate that sensor drifts are indeed a minor issue for CTD-SRDL.

Adjustments parameters were estimated for each CTD-SRDLs separately by comparisons of salinity measurements with available data in the World Ocean Database^35^. Because the southern ACC region (south of 55°S) is associated with a large-scale upwelling of circumpolar deep waters near the surface, the salinity at depth is very stable there, with a low natural variability highly suitable for use as a reference. Salinity data cross-comparisons between different CTD-SRDLs were also used to estimate suitable adjustments for CTD-SRDLs having no profiles available in the southern ACC region.

Once calibrated, the accuracy of post-processed CTD-SRDL measurements was estimated to be ±0.03 °C in temperature and ±0.05 psu (practical salinity unit) or better in salinity for CTD-SRDLs built after 2007^28^ (against ±0.01 °C and ±0.01 psu respectively for Argo profiles). The achieved accuracy is highly dependent upon availability of ship-based CTD comparisons, and the type of water masses sampled during the deployment time. In best cases, an accuracy of ±0.01 °C and ±0.02 psu can be obtained. However, no detailed estimation of post-processed uncertainty that is tag dependent has been attempted at this stage. The same uncertainty values are attributed to every CTD-SRDL profiles, except for pre-2007 CTD-SRDLs (about 5% of profiles) which used an older technology with a poorer accuracy roughly estimated around ±0.1 °C and ±0.1 psu.

## Data Records

The dataset comprises profiles of temperature (°C) and practical salinity (psu) as a function of pressure (dbar). Each profile is located in space and time. It must be emphasized that the dataset of each individual CTD-SRDL has been edited and corrected separately, as a given CTD-SRDL has its own specificities in terms of data accuracy and quality of the estimated correction (see above).

Data are provided following the Argo netCDF format^36^. The generic netCDF standard is a self-documented binary format developed specifically store geo-referenced climate data (more information at www.unidata.ucar.edu), which has become very popular to store hydrographic data because it can be read and manipulated with ease with a wide variety of data processing software programs and programming languages. The Argo netCDF format is a standard used to store Argo float data on Argo data servers, and thus it is well adapted to record hydrographic profile data. The Argo netCDF format includes a complete set of metadata for each profile, with data quality flags, the possibility to record both raw and adjusted data values, and the ability to record applied calibration equations. Most significant variables are shown in Table 2.

While files in Argo netCDF format are meant to be the reference data files, data are also provided in Ocean Data View (ODV) spreadsheet format. Ocean Data View is a cross-platform software designed to manipulate and visualize ocean data^37^. The ODV spreadsheet format is an ASCII format, which means that any text editor can read it. Contrary to the Argo netCDF files, ODV spreadsheet files contains only adjusted values that have been flagged as good data. Also, the amount of metadata in the ODV spreadsheet files is kept to a minimum, i.e., SMRU name, Julian date, location, and pressure/temperature/salinity data.

The Southern Indian Ocean seal subset is available to the public through an unrestricted repository at the BODC portal (Data Citation 1).

## Technical Validation

The surface dynamic height anomaly relative to a given reference pressure level is obtained by vertically integrating inverse density anomalies^38^. It is a key quantity in physical oceanography because its horizontal gradient is directly proportional to the large-scale (geostrophic) currents if one assumes that currents are negligible at the reference pressure level. It is also a useful quantity to validate seal data, because it provides a single scalar quantity for each profile that depends on both temperature and salinity profiling data.

Here we consider the dynamic height anomaly at 20 dbar relative to 500 dbar (here denoted as DH500) to validate the seal database, comparing its spatial distribution based on raw and adjusted seal data with the distribution obtained using hydrographic profiles present in the World Ocean Database (WOD) for the period 2000 to 2013 (ref. 35). The choice of the reference level is a compromise between the number of seal profiles that are used in the validation procedure (a 400 dbar reference level would allow to use 80% of seal profiles, against 60% only for the 500 dbar reference level and 20% for 800 dbar, see Fig. 2b), and the amount of deep data used to compute dynamic height anomalies, as hydrographic data deeper than the reference level are not used to compute the dynamic height anomaly. The 500 dbar reference level is chosen because it approximately maximizes the proportion of data considered in the dynamic height calculation, as determined by the product of the reference depth with the number of retained profiles. Although ocean currents are generally not negligible at 500 dbar (i.e., around 500 m depth) in the Southern Ocean, the surface dynamic height anomaly relative to 500 dbar gives a good representation of the position and relative magnitude of ocean currents in the Southern Ocean^14^, because the vertical structure of ocean currents is highly coherent there^39^.

**Figure 3:**
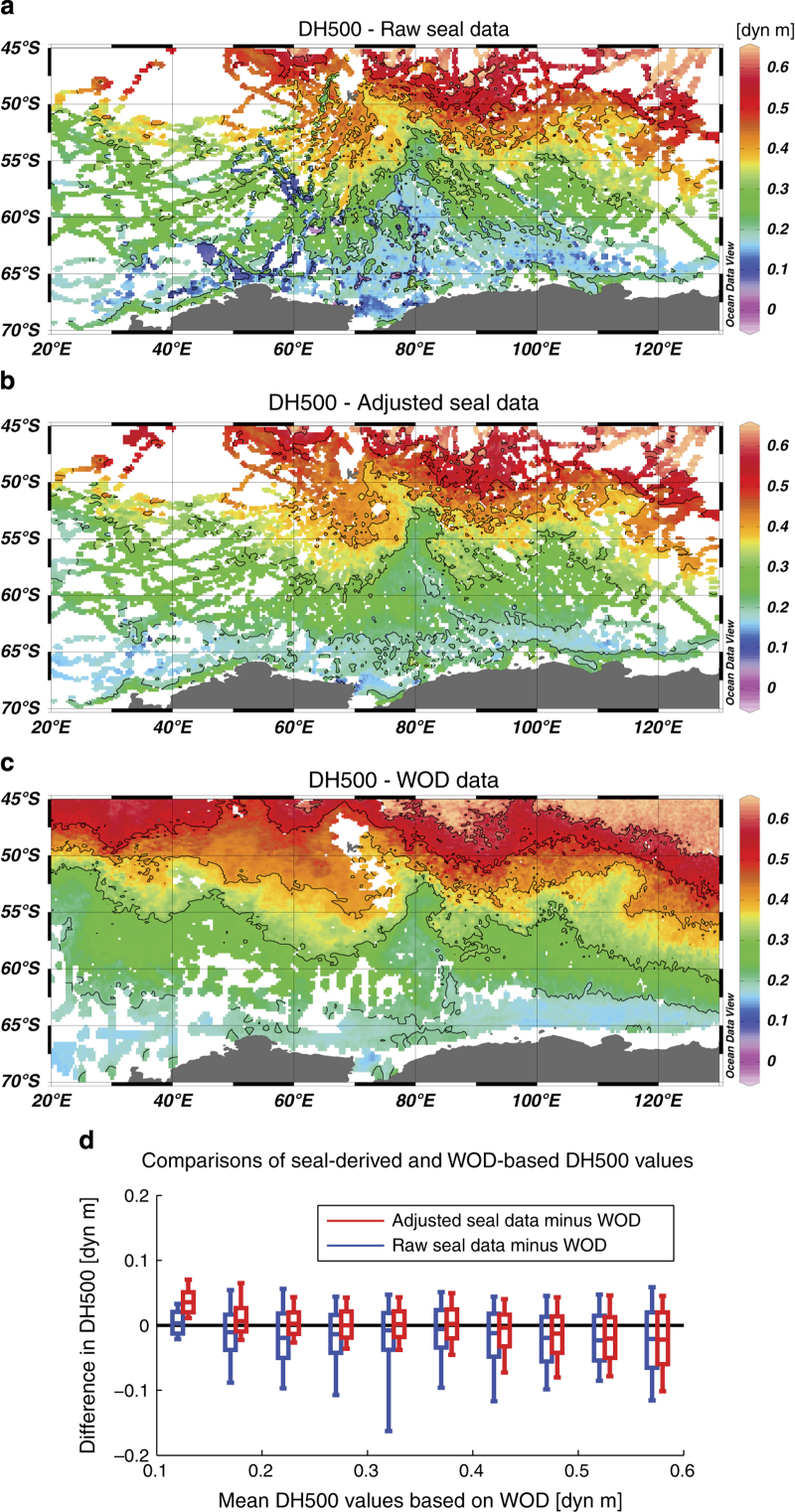
Validation of seal-derived hydrographic data. Comparisons between DH500 values (Dynamic Height Anomaly at 20 dbar relative to 500 dbar), obtained from (**a**) raw seal data, (**b**) adjusted seal data, and (**c**) the World Ocean Database (WOD^35^). (**d**) Mean and standard deviation of the differences with WOD-based DH500 values binned using a 0.05 dyn m interval are presented for both adjusted (red boxes) and raw (blue) seal data. On each box, the central mark is the median, while the edges of the box and whiskers are respectively the 16th and 3rd percentiles. Adjustments on seal data improve significantly comparisons with WOD data.

In our region of interest (20E–130E, south of 45S), a total of 42,300 hydrographic profiles are available in WOD, mostly comprised of Argo and ship-based CTD data, against 73,572 seal profiles (i.e., 73.9% more than other WOD profiles). Prior to the comparison, DH500 values are interpolated on a regular grid of resolution 1/3° lat × 1/6° lon, using the DIVA gridding tool available in ODV^37^. The mean difference between seal-based DH500 values and WOD-based values is of only −0.002±0.027 dyn m (mean±s.d.), against −0.015±0.041 dyn m for raw seal data. This is clearly reflected in DH500 maps (Fig. 3), showing that the DH500 distribution based on adjusted seal data resembles closely the distribution based on WOD data wherever seal data are available, and that adjustments improve significantly the comparison everywhere. One interesting exception is found in the southernmost part, where seal-based DH500 values are larger on average than WOD-based values. This is a result of the availability of seal data over the Antarctic continental shelf, where fresh waters are found. The lowest DH500 values are found at Cape Darnley, associated with very high salinity dense shelf water formed inside the Cape Darnley polynya^23^.

The data coverage is particularly improved by seal data over the northern Kerguelen Plateau (around 70°E, 50°S). There, the shallow bathymetry prevents Argo floats from entering the area resulting in a very low data density in WOD, while at the same time there is a lot of seal data available because it is a major feeding site for the animals. The mean difference and spread between seal and WOD-based DH500 values are larger in high-DH500 areas (>0.45 dyn m) characterising the subantarctic and subtropical zones. This is not surprising because there are few seal profiles available there, while these regions are most often characterized by large natural variability associated with eddies and frontal displacements of the ACC.

## Usage Notes

Instrumented seals are filling very important gaps in what is traditionally a very data-poor region of the world oceans, and therefore are becoming increasingly utilised by oceanographers studying the role of the Southern Ocean and Antarctica in global climate. Seal-derived data are making a growing contribution to climatologies built upon existing oceanographic databases, such as the World Ocean Database^35^. The previous deployments have established an important baseline and the time series will grow and with it the confidence to assess how the Southern Ocean is changing and why^40–42,40–42,40–42^. The accuracy of CTD-SRDL hydrographic data is also expected to increase significantly in the coming decade, as the technology improves. This will be very useful to assess ocean changes such as those associated with the melting of the Antarctic ice sheet and changing sea ice distribution, as these issues require a high level of accuracy for salinity data (ideally ±0.01 psu) that is only marginally achieved so far.

It is important to monitor the distribution and variations of hydrographic properties of seawater because they control the local density of seawater, and in turn, the distribution of geostrophic currents, i.e., the large-scale component of ocean circulation resulting from a balance between the horizontal pressure gradient and Coriolis forces. The distribution of temperature and salinity near the ocean surface is a particularly critical climate factor, as it is a result of complicated air-sea-ice interactions that can feedback on the atmospheric circulation. Instrumented seals provide an important contribution to the Southern Ocean Observing System (SOOS)^43^, which promotes the coordination of the different observing technologies, including *in situ* cruises, floats, drifters, satellites, etc. Regional and global climate models will directly benefit through the augmented observational datasets at their disposal for evaluating their performance, which in turns leads to better predictions and advise from the climate science community to government and business managers.

Seal-based hydrographic data can also be used to produce improved state estimates of the ocean circulation^12^. The satellite remote sensing community estimating sea ice production, drift velocity and fast ice mapping will utilise seal data to evaluate and validate their products^11^. Recently, it has become possible to add a fluorometer on CTD-SRDLs, which allows collecting vertical profiles of chlorophyll concentration together with hydrographic profiles^44,45^. This technology is quickly becoming a major source of information on the primary production in the iron-limited Southern Ocean. The seal dataset is also contributing to our understanding of the use of oceanographic features by foraging southern elephant seals in the Southern Ocean, and more particularly in the Kerguelen Plateau region, and is now successfully used to improve model-derived estimates of animal movements.

## Additional information

**How to cite this article:** Roquet, F. *et al.* A Southern Indian Ocean database of hydrographic profiles obtained with instrumented elephant seals. *Sci. Data* 1:140028 doi: 10.1038/sdata.2014.28 (2014).

## Supplementary Material



Supplementary Figure 1

## Figures and Tables

**Table 1:** Summary information on seal deployments in the Southern Indian Ocean.

**Deployment site**	**#tag**	**Period**	**#TS profiles**	**#/day**	**#data/profile**
Kerguelen Isl.	143	2004–2013	39826	2.45	17.2
Davis Station	42	2011–2012	25014	4.01	18.0
Casey Station	22	2012	11872	3.27	15.4
Total	207	2004–2013	76704	2.94	16.5

**Table 2:** Important variables in netCDF Argo data files^36^.

**Name**	**Dimension**	**Quick description**
SMRU_NAME	N_PROF	CTD-SRDL unique identifier
PI_NAME	N_PROF	Principal Investigator name
JULD_LOCATION	N_PROF	Julian date since 1950-01-01
LATITUDE	N_PROF	Estimated latitude
LONGITUDE	N_PROF	Estimated longitude
PRES	N_PROF x N_LEVELS	Raw Pressure
PRES_ADJUSTED	N_PROF x N_LEVELS	Adjusted pressure
PRES_ADJUSTED_QC	N_PROF x N_LEVELS	Pressure flag (1: good quality)
TEMP	N_PROF x N_LEVELS	Raw temperature
TEMP_ADJUSTED	N_PROF x N_LEVELS	Adjusted temperature
TEMP_ADJUSTED_QC	N_PROF x N_LEVELS	Temperature flag (1:good quality)
PSAL	N_PROF x N_LEVELS	Raw practical salinity
PSAL_ADJUSTED	N_PROF x N_LEVELS	Adjusted practical salinity
PSAL_ADJUSTED_QC	N_PROF x N_LEVELS	Practical salinity flag (1: good quality)
SCIENTIFIC_CALIB_COEFFICIENT	N_PROF x 2	Coefficients of calibration used to obtain adjusted data for T and S
