# Supplementary material for: A Southern Indian Ocean database of hydrographic profiles obtained with instrumented elephant seals
Source: Sci Data. 2014 Sep 2;1:140028. doi: 10.1038/sdata.2014.28 (PMC4322578; doi:10.1038/sdata.2014.28)
Supplement: Supplementary Figure 1 [file sdata201428-s2.pdf]

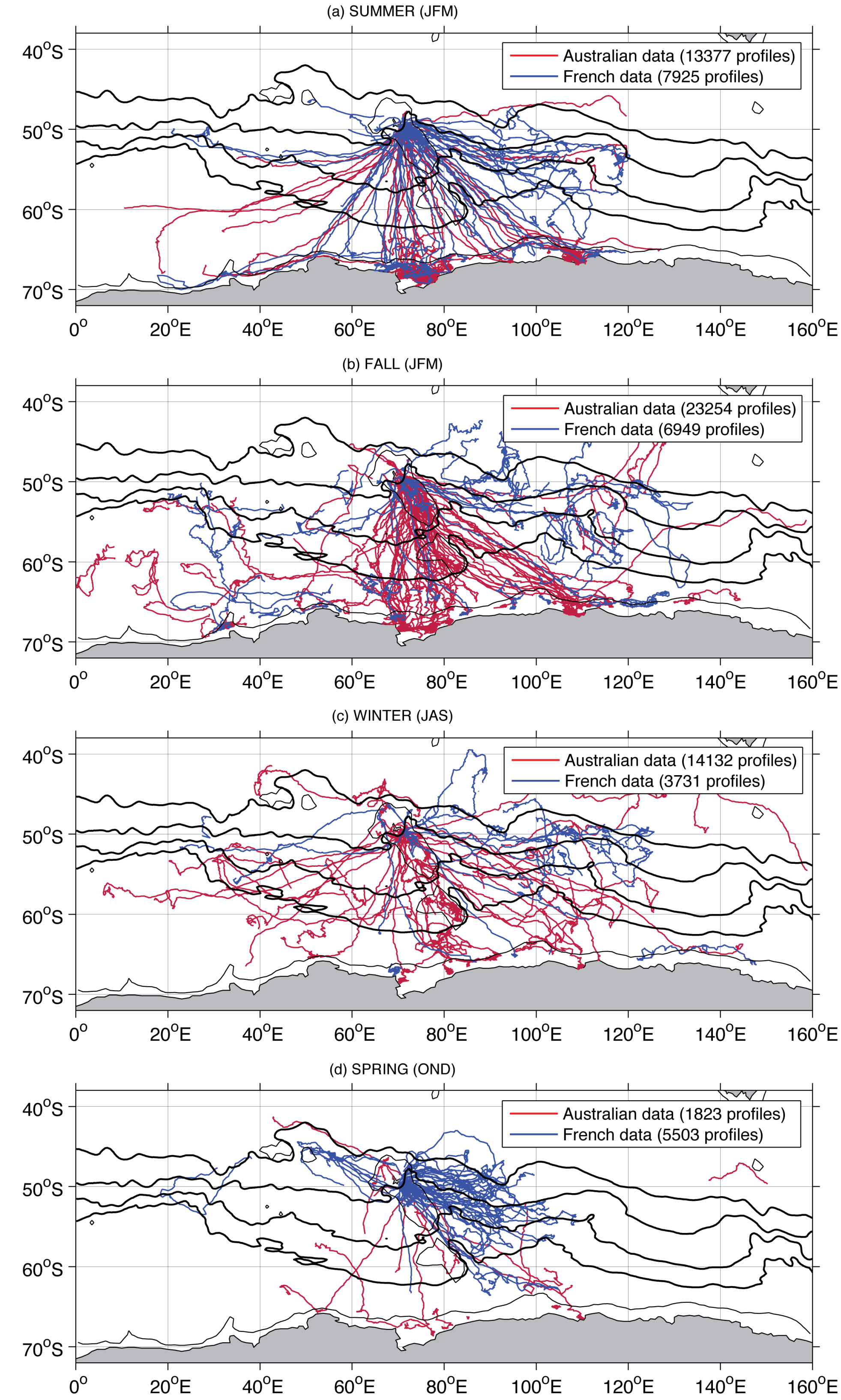


**Supplementary figure S1: Seasonal distribution of seal-derived profiles in the Southern Indian Ocean database.** Same as in Fig. 1a, but for each austral season separately.
